# Randomized controlled study to evaluate the safety and clinical impact of percutaneous auricular vagus nerve stimulation in patients with severe COVID-19

**DOI:** 10.3389/fphys.2023.1223347

**Published:** 2023-08-08

**Authors:** Tamara Seitz, Franziska Bergmayr, Reinhard Kitzberger, Johannes Holbik, Alexander Grieb, Julian Hind, Felix Lucny, Alexander Tyercha, Stephanie Neuhold, Claus Krenn, Christoph Wenisch, Alexander Zoufaly, Eugenijus Kaniusas, József Constantin Széles

**Affiliations:** ^1^ Department of Infectious Diseases and Tropical Medicine, Clinic Favoriten, Vienna, Austria; ^2^ Faculty of Medicine, Sigmund Freud University, Vienna, Austria; ^3^ Department of General Surgery, Division of Vascular Surgery, Center for Wound Surgery and Special Pain Therapy, Health Service Center of Vienna Privat Clinics, Medical University of Vienna, Vienna, Austria; ^4^ Department of Anesthesiology and General Intensive Care Medicine, Medical University of Vienna, Vienna, Austria; ^5^ Department of Orthopedics and Trauma Surgery, Medical University of Vienna, Vienna, Austria; ^6^ Faculty of Electrical Engineering and Information Technology, Institute of Biomedical Electronics, Vienna University of Technology (TU Wien), Vienna, Austria

**Keywords:** nervus vagus stimulation, auricular vagus nerve stimulation, COVID-19, ARDS, hyperinflammation

## Abstract

**Introduction:** A severe course of COVID-19 is characterized by a hyperinflammatory state resulting in acute respiratory distress syndrome or even multi-organ failure along a derailed sympatho-vagal balance.

**Methods:** In this prospective, randomized study, we evaluate the hypothesis that percutaneous minimally invasive auricular vagus nerve stimulation (aVNS) is a safe procedure and might reduce the rate of clinical complications in patients with severe course of COVID-19. In our study, patients with SARS-CoV-2 infection admitted to the intensive care unit with moderate-to-severe acute respiratory distress syndrome, however without invasive ventilation yet, were included and following randomization assigned to a group receiving aVNS four times per 24 h for 3 h and a group receiving standard of care (SOC).

**Results:** A total of 12 patients were included (six in the aVNS and six in the SOC group). No side effects in aVNS were reported, especially no significant pain at device placement or during stimulation at the stimulation site or significant headache or bleeding after or during device placement or lasting skin irritation. There was no significant difference in the aVNS and SOC groups between the length of stay in the intensive care unit and at the hospital, bradycardia, delirium, or 90-day mortality. In the SOC group, five of six patients required invasive mechanical ventilation during their stay at hospital and 60% of them venovenous extracorporeal membrane oxygenation, compared to three of six patients and 0% in the aVNS group (*p* = 0.545 and *p* = 0.061).

**Discussion:** Vagus nerve stimulation in patients with severe COVID-19 is a safe and feasible method. Our data showed a trend to a reduction of progression to the need of invasive ventilation and venovenous extracorporeal membrane oxygenation which encourages further research with larger patient samples.

## 1 Introduction

The COVID-19 pandemic, caused by SARS-CoV-2, has been ongoing for more than 2 years now, and the clinical presentation of the disease is known to vary widely. Although most cases of COVID-19 are mild or asymptomatic (Huang et al., 2020), there are some cases where the initial stage of viral replication is followed by a stage of hyperinflammatory response to SARS-CoV-2 infection resulting in severe disease with acute respiratory distress syndrome (ARDS) or even multi-organ failure (Fajgenbaum & June 2020). A hypothesized cause for developing such complications is a derailed sympathy-vagal balance (Guo et al., 2020; Zhou et al., 2020). More specifically, the infection leads to an overshooting response of the pro-inflammatory pathway of the sympathetic nervous system, while the modulating anti-inflammatory pathway of the parasympathetic nervous system is impaired.

The hyperactivity of the sympathetic nervous system in COVID-19 leads to an excessive release of epinephrine and norepinephrine, increasing pulmonic vasoconstriction and capillary permeability (Poulat and Couture, 1998; Busl and Bleck, 2015; Liu et al., 2017; Kaniusas et al., 2020a). Due to the pulmonary damage, the sympathetic nervous system is stimulated, further leading to an excessive release of pro-inflammatory cytokines, like IL-6. This cytokine storm induces pulmonic hemorrhage, edema, and atelectasis resulting in ARDS. Furthermore, the hyperactivity of the sympathetic nervous system aggravates inflammation through the renin–angiotensin–aldosterone system by releasing a cascade of vasoactive peptides (Vaduganathan et al., 2020), further exacerbating ARDS (Busse et al., 2020).

Moreover, the sympathy-vagal imbalance creates a state of severe hypoxemia and increases the risk for thrombotic or thromboembolic events possibly leading to acute myocardial injury and even chronic damage to the cardiovascular system (Guan et al., 2020; Zheng et al., 2020).

One theory suggests that the stimulation of the vagus nerve, the major nerve of the parasympathetic nervous system (Pavlov and Tracey, 2012), can decrease the sympatho-vagal imbalance while simultaneously increasing the anti-inflammatory activity of the parasympathetic nervous system.

Recent studies with vagus nerve stimulation showed clinical improvements in patients suffering from depression (Ferstl et al., 2021), epilepsy (Amar et al., 2022), rheumatoid arthritis (Staats and Poree, 2021), chronic back pain (Sator-Katzenschlager et al., 2004), and development of arrhythmia (Stavrakis et al., 2015). Animal experiments demonstrated a reduced production of inflammatory cytokines (Dos Santos et al., 2011; Krzyzaniak et al., 2011; Akella and Deshpande, 2015), a release of NO resulting in pulmonic vasodilation (Olshansky et al., 2008; Brack et al., 2009), and increased cerebral microcirculation (Schweighöfer et al., 2016).

Kaniusas et al. (2020b) suggested that auricular vagus nerve stimulation (aVNS) might have a positive impact in patients with severe COVID-19 through the activation of anti-inflammatory pathways, balancing of the sympathy-vagal ratio, and, therefore, reducing the follow-up risk of ARDS development as well as further complications. To diminish the side effects and ease application, percutaneous minimally invasive aVNS was suggested for patients, where miniature needle electrodes are positioned in the ear regions innervated mainly by the vagus nerve.

Here, we present a prospective and randomized study aiming to evaluate the safety and feasibility of aVNS in patients with moderate-to-severe ARDS without the current need of invasive ventilation. Furthermore, the clinical benefit is evaluated.

The main hypothesis is that the use of aVNS in patients with severe COVID-19 is well tolerated and safe, defined as follows:

a) No significant pain at device placement or during stimulation at the stimulation site
b) No significant headache
c) No bleeding after or during device placement
d) No lasting skin irritation (>1 h)
e) No increased risk of death
f) No increased risk of delirium
g) No increased risk of bradycardia (defined as heart rate lower 50 beats per minutes)
h) No increased length of stay (LOS)

Further hypotheses are that the use of aVNS in patients with severe COVID-19 reduces the need for and the duration of invasive mechanical ventilation.

The study design is published in Frontiers, 2022 (Seitz et al., 2022).

## 2 Materials and methods

### 2.1 Design

The study is a randomized controlled trial, open and not blinded. Since stimulation is perceived as a tingling sensation, placebo (with deactivated aVNS) was not considered as a reasonable option for blinding. A sham stimulation (of non-vagal regions such as the ear lap) was also not used as a control group, since the number of included patients with severe COVID-19 was relatively small.

### 2.2 Patient recruitment

All patients admitted to the intensive care unit (ICU) of the Department of Infectious Diseases and Tropical Medicine, Clinic Favoriten, Vienna, Austria, due to COVID-19 were screened by the attending physician regarding inclusion and exclusion criteria. During the time period of this study, only patients with a positive RT-PCR test for SARS-CoV-2 were admitted to this ICU. Acute respiratory failure with a PaO2/FiO2 lower than 200 mmHg requiring non-invasive ventilation (NIV) was necessary for enrolment (see Supporting Material S1 for detailed criteria).

If all inclusion criteria and none of the exclusion criteria were met, the attending physician informed the patient about the study. After written consent was obtained, randomization of the study group into the aVNS group (aVNS) or the standard of care (SOC) group was performed with a computer-based randomization tool.

### 2.3 Procedure

Participants assigned to the aVNS group immediately underwent aVNS. AuriStim (Multisana GmbH, Austria) was the device chosen to conduct the procedure, as shown in Supplementary Material Figure 1.

**FIGURE 1:**
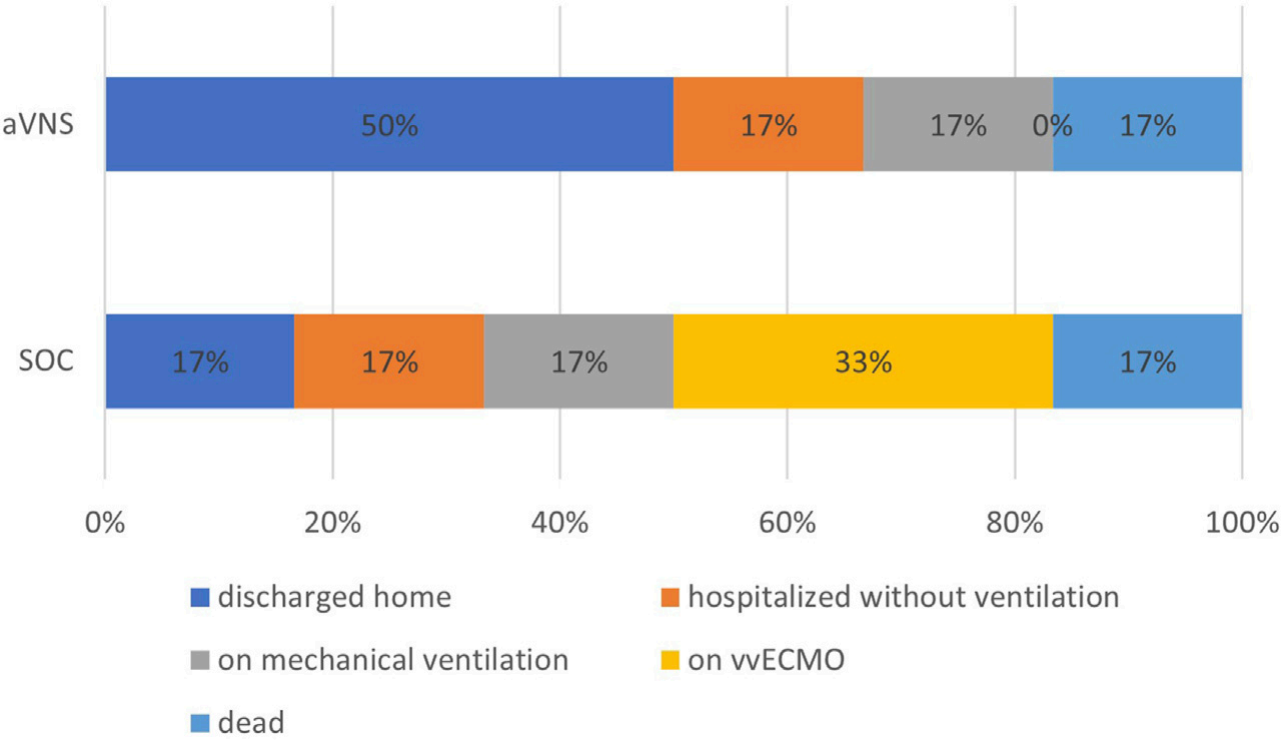
Clinical outcome of the participants of the SOC and aVNS groups at day 28 after study inclusion. SOC = standard of care and aVNS = auricular vagus nerve stimulation.

As a battery-powered single-use, miniaturized electrical stimulator, AuriStim delivers monophasic varying polarity pulses (pulse width of 1 ms) with a fixed amplitude (3.8 V), every second (stimulation frequency is 1 Hz), and a duty cycle (3 h ON/3 h OFF). The needle and needle-to-tissue interface resistance is about 4–7 kOhm with a resulting peak current at approximately 0.5–0.9 mA, remaining at or just below the recommended limits of about 1.5 mA.

Multi-punctual percutaneous aVNS was mediated through the usage of three miniature needle electrodes. These were inserted into vitally (solely or partly) innervated regions of the auricle (cymbal concha), the cavity of concha and the crura of antihelix (Peuker and Filler, 2002). The location of the needles was always in the vicinity of local blood vessels, which had been identified by transillumination of the auricle priory (Kaniusas et al., 2011), as well as the auricular nerves (Dabiri et al., 2020).

Once the needles were positioned, a fragmentary stimulation sequence alternating between 3 h of activity and 3 h of rest was started. Altogether, this added up to four cycles of 3 h of stimulation within 24 h. To prevent malfunction because of a potential battery drainage, the initial device was replaced every 3 days with a fully charged one during the treatment period. The procedure was carried out until the patient was transferred to another ward, discharged from the ICU, or died.

The visual analogue scale (VAS) was applied four times a day and during device placement to document any pain in the area, provided the patient was responsive at this time. The VAS is an easy-to-use tool to help patients subjectively define pain from a scale of 0 (no pain) up to 10 (worst possible, unbearable, excruciating pain). CAM-ICU was assessed two times a day, as long as the patient was responsive to screen for delirium. CAM-ICU is a tool to assess the presence of delirium in patients admitted to the ICU (Gusmao-Flores et al., 2012). Patients were specifically asked to distinguish between headache and pain at the stimulation site. Stimulation was immediately terminated should VAS have reached rates above 5. Regardless, patients always had the option of discontinuing stimulation due to discomfort.

Further potential side effects of aVNS were assessed by clinically examining and interviewing the patients twice a day. Particular attention was given to skin irritation or bleeding at the site of device placement. If skin irritation was present longer than 60 min, it was considered lasting.

As in the case of aVNS stimulation, the collection of clinical parameters took place while the patient was in the ICU until discharge or death. A subsequent follow-up 14, 28, and 90 days since study enrolment was performed.

### 2.4 Statistical analysis

Basic characteristics of the participants were collected, including age, gender, virus variant, time since symptom onset, comorbidities, and respiratory situation. This was a pilot study which meant a power analysis could not be conducted beforehand. Limited financial resources allowed the inclusion of 12 patients at total.

Categorical variables were described by means of absolute numbers and percentages. For continuous variables, the normal distribution of values within aVNS and SOC was investigated using the Shapiro–Wilk test. Parameters whose values were complied with a normal distribution were described with the mean and standard deviation, and comparisons between the groups were conducted with independent-sample t-tests. Welch correction was considered in case of heterogeneous variances. Parameters whose values were not normally distributed were described with the median and interquartile range (IQR). Group differences with respect to these parameters were performed with the Mann–Whitney exact test. Risk ratios with 95% confidence intervals were reported. The analyses were conducted with IBM SPSS Statistics. An alpha level of 5% was used for inferential statistics.

### 2.5 Ethical considerations

The study was approved by the local ethics committee (Ethikkommission der Stadt Wien - EK 21-079-0521) and Austrian Federal Office for Safety in Healthcare BASG. The study was registered at ClinicalTrials.gov (NCT05058742, 25/09/2021).

## 3 Results

At total of 12 patients were included (six in the aVNS group and six in SOC group). No patient was excluded later after inclusion in the study.

The basic parameters of the participants are listed in Table 1, and the clinical parameters of the participants at the time of study inclusion are listed in Table 2. There were no significant differences regarding basic or clinical parameters in patients of group aVNS and SOC.

**TABLE 1:** Basic parameters of patients in the aVNS and SOC groups. SOC = standard of care and aVNS = auricular vagus nerve stimulation.

	aVNS (*n* = 6)	SOC (*n* = 6)	*p*-value
Age (in years)			
Mean	53.8	52.7	*p* = 0.82
Standard deviation	9.7	8	
Gender, % (n)			
	33% female (2)	50% female (3)	*p* = 1
	67% male (4)	50% male (3)	
Comorbidities, % (n)			
Hypertension	67% (4)	50% (3)	*p* = 1
Obesity	67% (4)	67% (4)	*p* = 1
Median BMI	32.4	33.1	*p* = 0.97
Diabetes mellitus	50% (3)	67% (4)	*p* = 0.57
Chronic artery disease	17% (1)	0% (0)	*p* = 1
Chronic renal failure	17% (1)	17% (1)	*p* = 1
Chronic lung disease	17% (1)	0% (0)	*p* = 1
Thyroid disease	0% (0)	33% (2)	*p* = 0.46
Hematological disease	0% (0)	0% (0)	-p = 1
Rheumatological disease	0% (0)	17% (1)	*p* = 1
Organ transplant	0% (0)	17% (1)	*p* = 1
Current smoking	17% (1)	0% (0)	
SAPS II			
Mean	20.3	21.7	*p* = 0.60
Standard deviation	2.7	5.4	
SARS-CoV-2 vaccination % (n)	0% (0)	0% (0)	-

**TABLE 2:** Clinical parameters of patients in the aVNS and SOC groups. SOC = standard of care and aVNS = auricular vagus nerve stimulation.

	aVNS (*n* = 6)	SOC (*n* = 6)
Symptoms % (n)		
Fever	83% (5)	67% (4)
Coughing	83% (5)	50% (3)
Dyspnea	67% (4)	50% (3)
Malaise	33% (2)	50% (3)
Diarrhea	50% (3)	0% (0)
Anosmia	17% (1)	17% (1)
Sore throat	17% (1)	0% (0)
Cephalea	17% (1)	0% (0)
Virus variant % (n)		
B.1.617.2 (Delta)	83% (5)	100% (6)
unknown	17% (1)	0% (0)
Others	0% (0)	0% (0)
Time between the symptom onset and study inclusion (in days)		
Median	8.5	8.5
Minimum–maximum	9-17	5-22
Interquartile range	3	8
Inflammation parameters at the time of study inclusion		
Mean CRP (mg/dL)	152.2	86.9
Median IL-6 (pg/mL)	51	45
Mean fibrinogen (g/L)	4.4	4.1
Median ferritin (µg/L)	753	295.5
Median DDIMER (µg/mL)	1.5	1.5
Therapy, % (n)		
Corticosteroids	100% (6)	100% (6)
Other immunomodulating therapy	0% (0)	17% (1)
Remdesivir	33% (2)	67% (4)
Antimicrobial therapy	83% (5)	83% (5)
Horrowitz index at the time of ICU admission		
Mean	121.3	150.3
Standard deviation	33.7	60.1
Rox index at the time of ICU admission		
Mean	4.6	4.7
Standard deviation	0.6	0.9
Horrowitz index at the time of study inclusion		
Mean	115.83	107
Standard deviation	43.2	41.6
Time since study inclusion to intubation (in days)		
Mean	5	1.2
Standard deviation	6.3	0.8

### 3.1 Tolerance and safety

#### 3.1.1 Pain or discomfort at the stimulation site

The mean VAS at the time of device placement was 3.2 (range 2-5). The mean VAS during stimulation was 1.8 (range 0-3). No patient opted to terminate aVNS prematurely.

#### 3.1.2 Headache

One patient in the aVNS group was suffering from headache at hospital admission, prior to the start of aVNS. The pain improved during hospital stay. Therefore, no connection between aVNS and headache, in this case, can be assumed. One patient in the SOC group reported headache during hospital stay responding well to analgesic therapy.

#### 3.1.3 Bleeding or skin irritation

There was no case of bleeding or skin irritation at the stimulation site.

#### 3.1.4 Bradycardia

There was no case of bradycardia reported.

#### 3.1.5 Delirium

There was no case of delirium in the VNS group. In comparison, there were two cases of new delirium in the SOC group.

#### 3.1.6 LOS and mortality

The results of LOS at the ICU and hospital in general, as well as mortality rates, are seen in Table 3. The mean duration of ICU LOS of the eight patients who were discharged was 18.9 days, in the SOC group 25.5 days and in the aVNS group 12.3 days. The difference is not significant (*p* = 0.195). There was no significant difference in LOS at hospital (*p* = 0.139) as well. There was no difference in 28-day-mortality or 90-day-mortality.

**TABLE 3:** Respiratory parameters in participants in the aVNS and SOC groups. SOC = standard of care and aVNS = auricular vagus nerve stimulation.

	aVNS	SOC	Risk ratio [95% CI]
Need of invasive ventilation, % (n)	50% (3)	83% (5)	0.6 [0.250–1.442]
vvECMO, % (n)	0% (0)	67.7% (4)	0.1 [0.007–1.699]
28 days mortality, % (n)	17% (1)	17% (1)	1 [0.080–12.558]
90 days mortality, % (n)	17% (1)	50% (3)	0.5 [0.060–4.153]

	aVNS	SOC	*p*-value
Duration of invasive ventilation (in days)			*p* = 0.195
Mean	22	27.3	
Standard deviation		15.5	
Total length of stay at the ICU (in days)			*p* = 0.195
Mean	12.2	25.5	
Standard deviation	9.4	15.6	
Total length of stay at hospital (in days)			*p* = 0.139
Mean	18.8	43.5	
Standard deviation	11.2	26.8	

### 3.2 Respiratory failure and ventilatory support

At the time of admission to the ICU, all patients received high flow oxygen therapy first. The first measured mean Rox index, including SpO2, FiO2, and respiratory rate, was 4.7, and the mean Horowitz index (PaO2/FiO2 ratio) was 135.8. All patients needed NIV later. The mean Horrowitz index at the time of study inclusion was 111.4. As shown in Table 3, a total of 67% of the participants required intubation and mechanical ventilation; however, differences between the SOC and aVNS groups can be observed.

### 3.3 Clinical outcome

The clinical outcome was evaluated at day 14 and 28. The clinical outcome at day 14 is shown in Supplementary Material and for day 28 is shown in Figure 1.

## 4 Discussion

In this study, we demonstrated that the minimal-invasive aVNS has the potential to reduce COVID-19-associated complications and improves clinical outcome. Especially, the impact on the respiratory system is impressive as the use of aVNS seems to lessen the severity of COVID-19 ARDS.

ARDS is the most common complication of severe COVID-19 and described in up to 42% of all patients with COVID-19 pneumonia (Wu et al., 2020). The mortality ranges between 26% and 61.5% if admitted to a critical care setting. The mortality rises from 65.7% to 94% if the patient undergoes invasive ventilation (Wu et al., 2020).

A trend to a reduced risk of progression of ARDS and a shorter length of hospital stay was seen. In patients receiving SOC, 83% required invasive ventilation and later 80% of them required vvECMO due to progressive respiratory failure. In comparison, study participants who received aVNS needed invasive ventilation only in 50%, and none of them received a vvECMO. The mean stay at the ICU of patients receiving aVNS was shorter by one-third compared to patients in the SOC group (9 *vs.* 28.5 days). The differences were not statistically significant, but this may be due to the small sample size. The calculated risk ratio for the need of mechanical ventilation in patients receiving aVNS was 0.6 compared to those without aVNS. In comparison, in a randomized trial including patients with moderate and severe ARDS, the risk ratio of the use of corticosteroids was calculated as 0.66 (Angus et al., 2020).

We believe that aVNS might have led to an activation of anti-inflammatory pathways resulting in reduced hyperinflammation and cytokine release. This hypothesis is supported by one of our prior studies (Seitz et al., 2022) including some of these study participants where we measured inflammatory parameters throughout the clinical course. We were able to show that patients receiving aVNS displayed a significantly stronger decrease of serum levels of interleukin 6 (IL-6) and tumor necrosis factor (TNF) alpha level over time compared to patients receiving none. Boezaart et al. also reported two cases of patients with severe COVID-19 receiving transcutaneous aVNS in addition to SOC, which led to a rapid decrease of IL-6 (Boezaart and Botha, 2021). A correlation with worse clinical outcome in COVID-19 and high levels of IL-6 and TNF alpha has previously been reported (Del Valle et al., 2020). One explanation is that the excessive release of such cytokines recruits activated macrophages and T cells to the site of infection inducing local inflammation. Local tissue damage may follow, resulting in the destruction of lung parenchyma which eventually leads to ARDS and multi-organ failure (Tay et al., 2020). Furthermore, we demonstrated (Seitz et al., 2022) that non-specific inflammation biomarkers, such as c-reactive protein (CRP) and ferritin decreased while receiving aVNS. In comparison to patients who were not treated with aVNS, CRP and ferritin levels increased. Recent studies indicate that on one hand, severely elevated CRP and ferritin can worsen clinical outcomes in COVID-19 (Caricchio et al., 2021), and on the other hand, a decrease of inflammation parameters throughout the clinical course reduces the risk of development and severity of ARDS (Wu et al., 2020).

The method was considered safe as there were no significant side effects seen, especially no significant pain or skin irritation or bleeding at the stimulation site. There was no case of new headache, bradycardia, or development of delirium seen in patients with aVNS in our study.

### 4.1 Limitations and strength

We acknowledge that the biggest limitation is the small number of participants. Regardless, we would like to emphasize that this is a controlled and randomized pilot study and should animate the performance of further studies with larger numbers of participants to evaluate whether the effects are truly statistically significant. Another limitation is that the study is not blinded given that we were not able to find a technical solution to do so. However, one of the strengths of the study is the computer-based randomization. There was no difference regarding the basic or clinical and inflammation parameters between the two groups. A further strength is the long follow-up period.

## 5 Conclusion

To summarize, this study demonstrates that aVNS was well tolerated and no serious events were reported. A trend to a reduction of the rate and the severances of respiratory complications in critically ill patients suffering from severe COVID-19 was seen. It may present a viable option for additional treatment in future. We believe that aVNS might have an impact not only on COVID-19 but also in other hyperinflammatory diseases. Therefore, further research on this topic is needed.

## Data Availability

The original contributions presented in the study are included in the article/Supplementary Material. Further inquiries can be directed to the corresponding author.
