# Supplementary material for: Randomized controlled study to evaluate the safety and clinical impact of percutaneous auricular vagus nerve stimulation in patients with severe COVID-19
Source: Front Physiol. 2023 Aug 8;14:1223347. doi: 10.3389/fphys.2023.1223347 (PMC10442574; doi:10.3389/fphys.2023.1223347)
Supplement: Supplementary file 1 [file DataSheet1.docx]

Supplementary Material

# Randomized-controlled study to evaluate safety and feasibility of percutaneous auricular vagus nerve stimulation in patients with severe COVID-19

**Tamara Seitz, Franziska Bergmayr, Reinhard Kitzberger, Johannes Holbik, Alexander Grieb, Julian Hind, Felix Lucny, Alexander Tyercha, Stephanie Neuhold, Claus Krenn, Christoph Wenisch, Alexander Zoufaly, Eugenijus Kaniusas and József Constantin Széles***

*** Correspondence:** Corresponding Author: .c.szeles@gmail.com

# Supplementary Data

Inclusion criteria (all criterion are needed for inclusion):

• Positive for SARS-CoV-2 by RT-PCR test (defined as a CT value less than 30)

• Acute respiratory failure requiring non-invasive respiratory support

• PaO2/FiO2 <200

Exclusion criteria (one criteria is sufficient for exclusion):

• Age <18 years

• Pregnancy (to be excluded using serum beta HCG in women of childbearing age)

• Signs of infection, eczema, or psoriasis at the application site

• Active malignancy

• Implanted cardiac pacemaker, defibrillator, or other active implanted electronic devices

• Patient unable to consent

• Heart rate <60 beats/min

• Known vagal hypersensitivity

• History of haemophilia

# Supplementary Figures and Tables

## Supplementary Figures


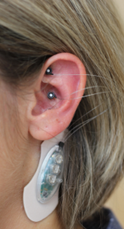

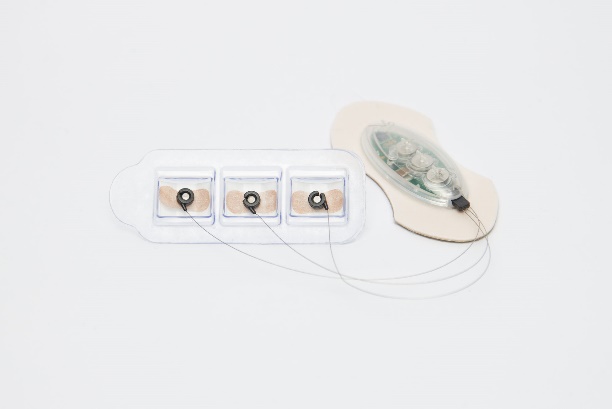


**Supplementary Figure 1.** Photograph of AuriStim (Multisana GmbH, Austria) a) prior to and b) during use.

**Supplementary Figure 2.** The clinical outcome of the participants of the SOC and aVNS group at day 14 after study inclusion.
